# In Situ Sputtering Silver Induction Electrode for Stable and Stretchable Triboelectric Nanogenerators

**DOI:** 10.3390/mi12101267

**Published:** 2021-10-18

**Authors:** Jinyuan Yao, Qi Zhang, Haodong Zhang, Mengqiu Li, Xichi Lu, Yu Xiao, Rujiao Yao, Xuhong Wang

**Affiliations:** 1 National Key Laboratory of Science and Technology on Micro/Nano Fabrication, Shanghai Jiao Tong University, Shanghai 200240, China; qzhang31@sjtu.edu.cn (Q.Z.); zhanghd0027@sjtu.edu.cn (H.Z.); li940619@sjtu.edu.cn (M.L.); xichi.lu@sjtu.edu.cn (X.L.); 2 Department of Micro/Nano Electronics, School of Electronic Information and Electrical Engineering, Shanghai Jiao Tong University, Shanghai 200240, China; 3 Institute of Spacecraft Equipment, Shanghai 200240, China; xiaoyush812@126.com (Y.X.); rjsh812@126.com (R.Y.); 4 Xirui Technology Co., Ltd., Jiaxing 314000, China; xuhong.wang@en-se.com

**Keywords:** triboelectric nanogenerator (TENG), in situ sputtering Ag, temporal stability, stretchable energy harvester

## Abstract

Triboelectric nanogenerators (TENG) can convert mechanical energy into electricity and exhibit unique advantages in the field of low-frequency and discrete energy harvesting. However, the interfacial state and stability between the triboelectric layer and electrode layer influence the output and applications of TENG. Herein, an in situ sputtering Ag process for fabricating induction electrodes is proposed to match with TENG. The sputtering Ag process is optimized by a variety of parameters, such as sputtering power, single-cycle time, number of cycles, cycle interval, and vacuum degree. In addition, the chemical state of Ag as a function of air placement is investigated, showing the sputtered Ag has excellent conductivity and stability. Moreover, four kinds of polymers are selected for fabricating TENGs based on the sputtered Ag induction electrodes, i.e., nylon 66, polyimide (PI), fluorinated ethylene propylene (FEP), and polydimethylsiloxane (PDMS), which shows great applicability. Considering the demand of flexible power suppliers, the sputtered Ag is integrated with a PDMS substrate, and shows good adhesion, flexibility, and ductility after severe deformation of the PDMS. Finally, the developed induction electrode processing technology is used in flexible TENG and shows great prospects in self-powered electronics for practical applications.

## 1. Introduction

To reduce the reliance on traditional fossil energy and realize the carbon-neutral environmental energy vision, it is urgent to develop new green and sustainable energy technologies all over the world [1,2,3,4,5]. Presently, energy sources such as solar, wind, ocean, and biomass energy have been developed and used [6,7,8,9,10,11,12]. However, in addition to these large-scale energy sources, our living environment is also scattered with a large number of irregular, low-frequency, discrete energy sources, such as raindrop, water-wave, and tiny mechanical energy [13,14,15,16,17,18]. These energy sources are small; however, the number is large, and if used effectively, they will make an important contribution to energy utilization.

Triboelectric nanogenerators (TENG), coupled with contact electrification and electrostatic induction, could convert mechanical energy surrounding the environment into electricity, and exhibit unique advantages in the field of low-frequency and discrete energy harvesting [19,20,21,22,23,24]. Usually, the core structure of a TENG is composed of the triboelectric layer and electrode layer. The triboelectric layer is used to generated polarized charges during the contact–separation movement, and the electrode layer is used to balance the potential difference in the electrostatic induction process. Additionally, considering the reliability of TENG, the excellent conductivity and stability are indispensable for the induction electrode.

Commonly, the methods of fabricating induction electrodes mainly include physical transfer printing [25] and conductive film sticking [26,27]. The electrode processed by physical transfer printing also suffer from issues such as poor stability over time and mechanical failure. For the electrode fabricated by sticking conductive film, such as copper and aluminum tape, the presence of polymer glue at the interface between the conductive tape and triboelectric layer will influence the electrostatic field distribution during the electrostatic induction process, thereby affecting the output of TENG. In addition, for induction electrodes fabricated by sticking or physical transfer, theoretically, there is a vacuum gap between the electrode layer and triboelectric layer, which may also cause adverse effects on the electrostatic induction. However, for the electrode fabricated by sputtering technology under a high vacuum, the conductive layer is in close contact with the friction layer, which could avoid the vacuum gap and improve the performance of TENG.

In this work, we report an in situ sputtering Ag process for fabricating induction electrodes used in TENG. The sputtering Ag process is optimized by a variety of parameters, such as sputtering power, single-cycle time, etc. In addition, the chemical state of Ag as a function of air placement is investigated, showing the sputtered Ag has excellent conductivity and stability. The sputtered Ag is also deposited on four dissimilar polymers and shows excellent compatibility and applicability. Considering the demand of flexible power suppliers, the sputtered Ag is integrated with a PDMS substrate, and shows good adhesion, flexibility, and ductility after the severe deformation of PDMS. Finally, the developed induction electrode processing technology is used in flexible TENG and shows great prospects in self-powered electronics for practical applications.

## 2. Materials and Methods

### 2.1. Optimization of the Sputtered Ag Process

This work used magnetron sputtering to fabricate Ag induction electrodes. The triboelectric layer of TENG is generally polymer, thus it may not be able to withstand the bombardment of high-power particle clusters. In addition, continuous bombardment on the surface of polymer for a long time will cause local surface temperature to rise and damage the surface of the triboelectric layer. Therefore, the in situ sputtering Ag electrode process was optimized by systematically investigating the sputtering power, single-cycle time, intermittent time, sputtering vacuum, and other parameters, so that it had a certain thickness, excellent conductivity, and good compatibility with polymer substrates. Specifically, the sputtering vacuum is related to the binding force between polymer substrate and sputtering Ag; the sputtering power is related to the amount of bombarded Ag per unit time, the single-cycle sputtering time and cycle interval control the surface temperature of the polymer substrate, and the cycle number guarantees the conductivity of the induction electrode. The optimized parameters used in this work are as follows: vacuum degree: 5 × 10^–4^ Pa, sputtering power: 150 W, single-cycle sputtering time: 90 s, cycle interval: 300 s, and cycle number: 6 times.

### 2.2. Fabrication and Measurements of the Dual-Electrode TENG

To verify the applicability of sputtered Ag electrodes, four different polymer substrates were selected for TENG assembly and output testing. A dual-electrode TENG was fabricated, polyethylene glycol terephthalate/indium tin oxide (PET/ITO) was selected as one of the triboelectric and electrode layers, the TENG output based on four triboelectric systems (nylon 66-PET, PI-PET (polyimide-PET), FEP-PET (fluorinated ethylene propylene-PET), and PDMS-PET (Polydimethylsiloxane-PET)) was tested, and the movement of TENG was controlled by a linear motor. A Keithley 6514 electrometer was used to complete signal acquisition, including short-circuit current, open-circuit voltage, and transfer charge.

### 2.3. Fundamental Characterization and Analysis

The micro-morphology of sputtered Ag, PET, ITO, nylon 66, PI, FEP, and PDMS were obtained on a JEOL JSM-7800F field-emission scanning electron microscope (JEOL Ltd., Beijing, China). The chemical states of sputtered Ag with and without air exposure were analyzed by X-ray photoelectron spectroscopy using a K-Alpha XPS (Thermo Fisher Scientific, Waltham, MA, USA). All X-ray photoelectron spectroscopy (XPS) measurements were performed at room temperature, the survey spectra of sputtered Ag were obtained, and the fitted components of Ag 3d were obtained by software. The phase structure of sputtered Ag was examined on a Rigaku D/max 2500 PC diffraction equipment (Rigaku, Tokyo, Japan) with Cu Ka radiation.

## 3. Results and Discussion

### 3.1. Stability Analysis of Sputtered Ag

**Figure 1:**
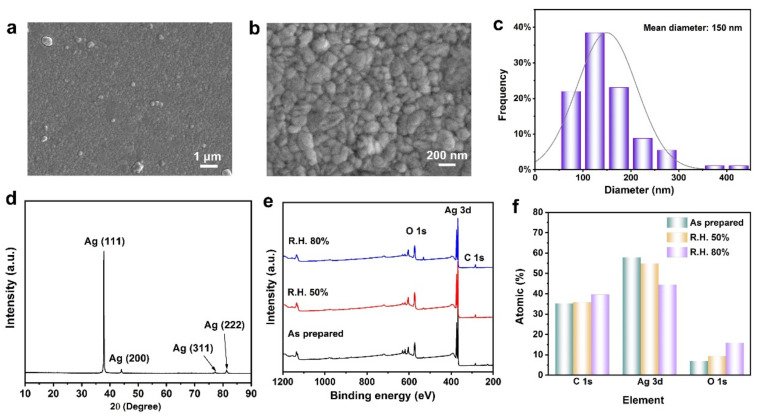
Characterization of the sputtered Ag. (**a**,**b**) The scanning electron microscopy (SEM) images of sputtered Ag with different magnifications; (**c**) Statistical analysis of the sputtered Ag size; (**d**) The X-ray diffraction (XRD) spectrum of Ag, including distinct characteristic peaks; (**e**) The survey X-ray photoelectron spectroscopy (XPS) of sputtered Ag, which is exposed at different ambient air and is mainly composited of C, O, and Ag; (**f**) The atomic percentage of sputtered Ag with different exposure conditions.

Figure 1a,b show the SEM images of sputtered Ag, indicating that the sputtered Ag film is flat. Typically, the Ag particles are uniformly and densely distributed, and there are obvious boundaries among the Ag particles. Figure 1c shows the size distribution of Ag, and the mean diameter is about 150 nm calculated from the Nano Measurer based on random statistics. However, there is a small number of particles with a larger size (about 400 nm), which may be attributed the agglomeration of particle clusters during the sputtering process. To confirm the crystal structure of sputtered Ag, X-ray diffraction (XRD) analysis was used, as shown in Figure 1d. The XRD diffraction spectrum shows four distinct characteristic peaks at 37.9°, 44.1°, 77.2°, and 81.2°, which correspond to the (111), (200), (311), and (222) crystal planes of face-centered cubic silver. In addition, the peak shape is sharp with excellent crystallinity and has obvious preferred orientation. To analyze the stability of sputtered Ag, X-ray photoelectron spectroscopy (XPS) was used to obtain the electronic structure and chemical state of Ag at different ambient pressures, as shown in Figure 1e. Survey XPS spectra showed that sputtered Ag film is mainly composed of Ag, C, and O, and there is no obvious difference with or without air exposure in different ambient pressures. Figure 1f is the atomic percentage of sputtered Ag film, showing the oxygen content increases after being exposed to the air.

**Figure 2:**
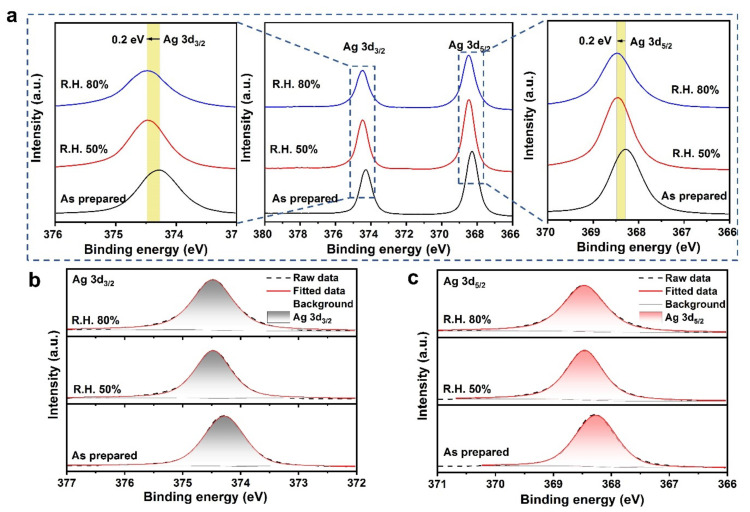
Detailed XPS spectra of Ag 3d photoelectron core level. (**a**) The detailed Ag 3d spectra with and without air exposure; (**b**) The fitted Ag 3d_3/2_ spectra with and without air exposure; (**c**) The fitted Ag 3d_5/2_ spectra with and without air exposure.

To examine the stability of sputtered Ag, the Ag films were placed in different relative humidity environments for 1 week, i.e., 50% R.H. and 80% R.H. As shown in Figure 2a, the peak position of Ag 3d has shifted after exposure in air, that is, it towards higher binding energy. For the binding energy of silver oxides (AgO and Ag2O), its binding energy is lower than that of metallic silver. Therefore, it can be concluded that the Ag is not oxidized in a large amount from the Ag 3d spectrum. For the sputtered Ag placed in 50% R.H. and 80% R.H., it is theoretically easier to react with water vapor than the sample as prepared. To further compare the chemical states of Ag film with and without air exposure, the Ag 3d_3/2_ and Ag 3d_5/2_ spectra were fitted. For both Ag 3d_3/2_ and Ag 3d_5/2_ (Figure 2b,c), the shape of the peaks is almost symmetrical and there is no splitting, indicating that no new composition is formed. However, compared with unexposed sputtered Ag, the binding energy of Ag is blueshifted by 0.2 eV after being placed in air for 1-week, which may be caused by measurement errors. Therefore, the sputtered Ag exhibits excellent stability, even in harsh environments, such as an 80% relative humidity ambient.

### 3.2. TENG Output Based on Sputtered Ag Induction Electrode

**Figure 3:**
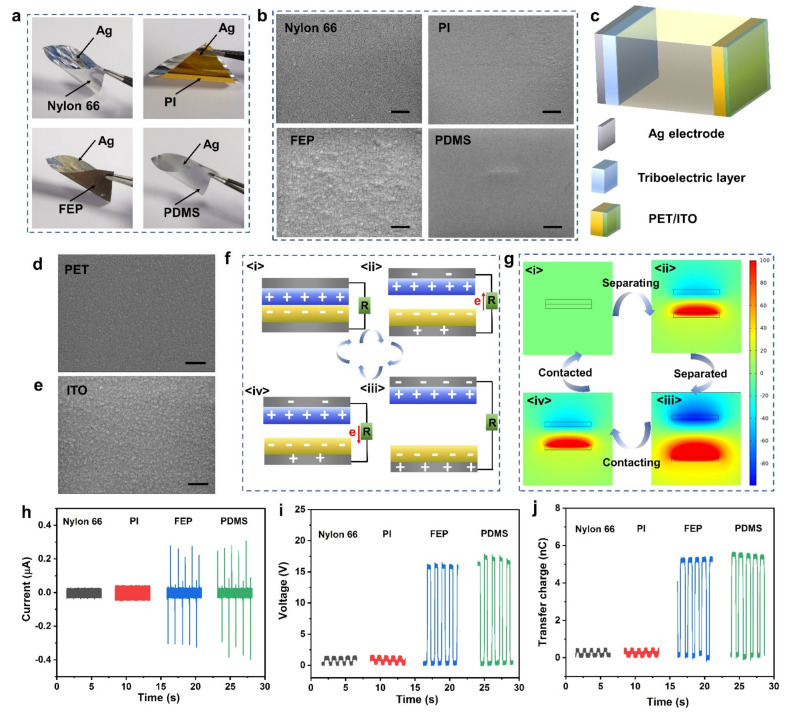
Triboelectric nanogenerator (TENG) output based on sputtered Ag induction electrode. (**a**) Optical images of different polymer films with the sputtered Ag; (**b**) SEM images of nylon 66, polyimide (PI), fluorinated ethylene propylene (FEP), and Polydimethylsiloxane (PDMS); (**c**) Schematic of the dual-electrode contact–separation TENG, the contact area of TENG is 4 cm^2^; (**d**,**e**) SEM images of polyethylene glycol terephthalate (PET) and indium tin oxide (ITO); (**f**) Schematic diagram of the working principle of TENG; (**g**) Electrostatic simulation of TENG during a periodic contact–separation movement; (**h**–**j**) The electrical signals of TENGs based on different triboelectric systems. The scale bars represent 500 nm.

To test the applicability of sputtered Ag induction electrodes for TENG, four kinds of polymers were selected, i.e., nylon 66, polyimide (PI), fluorinated ethylene propylene (FEP), and polydimethylsiloxane (PDMS), and then Ag was deposited on the polymer surface through an optimized sputtering process, as shown in Figure 3a. The four polymer substrates are all flexible, among them, PDMS has the best flexibility. The thickness of these polymer films is in the order of tens to hundreds of microns (Figure S1, Supplementary Materials), the sputtered Ag can be well-bonded to the polymer film, and the used sputtering process does not cause damage to the film (Figure S2, Supplementary Materials). Figure 3b includes the SEM images of polymer films, indicating that the surfaces are flat and different from each other (Figure S3, Supplementary Materials).

To verify the practicability of sputtered Ag, a contact–separation TENG was used for examination (Figure 3c, Figure S4, Supplementary Materials). This is a traditional dual-electrode model, using a commercial polyethylene glycol terephthalate (PET) film coated with indium tin oxide (ITO) as another electrode and triboelectric layer, respectively. Figure 3d,e is the SEM images of PET and ITO, showing the morphology is flat and dense. Compared with PET, nylon 66 is more prone to lose electrons, therefore, the nylon 66 is used as a positively triboelectric layer. While for FEP, PDMS and PI, PET is easier to lose electrons, so the FEP, PDMS, and PI are used as negatively triboelectric layer. Figure 3f shows the working mechanism of the contact–separation dual-electrode TENG, which is involved in contact electrification and electrostatic induction. When two materials at different positions in the triboelectric sequence come into contact, electron transfer occurs due to the difference in the ability of gaining or losing electrons. The identical amount of charges with opposite polarity are appeared at the interface of the dissimilar materials, and they are neutralized each other (Figure 3f(i)). Subsequently, when the dissimilar materials begin to separate, part of charges at the surface cannot be neutralized due to the presence of air layer. Therefore, an electrostatic field is generated inside the induction electrode on the back, causing charges separation and forming a current in the external circuit (Figure 3f(ii)). As the separation distance increases, the charges that cannot be neutralized at the dissimilar surface increase. Thus, the electrostatic field acting on the induction electrode increases, resulting in an increase in the separation charges and external circuit current (Figure 3f(iii)). Afterwards, when the dissimilar materials gradually approach, part of charges at the surface could neutralize each other. Therefore, the electrostatic field generated in induction electrodes is reduced, causing the reverse flow of electrons and forming a reverse current (Figure 3f(iv)). Figure 3g is the surface potential change of the TENG electrode in the contact–separation movement, which is obtained by the electrostatic field simulation. Detailly, give a certain charge density to the surface of two triboelectric layers, enter the boundary conditions of the electrostatic field and change the distance of the triboelectric layers to calculate the surface potential. When the dissimilar materials complete contact electrification, that is, the material surface has a certain charge density. The electrode surface potential increases with the increasing separation distance, and finally reaches the equilibrium. Figure 3h–j is the output characteristics of TENG based on different triboelectric systems, its movement is controlled by a linear motor, and the testing procedures is also added in Note S1 (Supplementary materials). Under the same test conditions, the electrical output based on different triboelectric materials is significantly different. Specifically, the output based on nylon 66 and PI is prominently lower than that of based on PDMS and FEP. The ability of triboelectric layer to gain or lose electrons will affect the surface charge density during the contact–separation process. The greater the surface charge density, the stronger the electrostatic field formed. Therefore, the more charges separated during the electrostatic induction process, the stronger the output of the TENG. Compared with nylon 66, PDMS is easier to obtain electrons. Therefore, it could produce a greater surface charge density when in contact with PET, accordingly, the output of the TENG is also larger.

However, based on the sputtered Ag electrodes prepared in this work, various TENGs have shown practical applicability.

### 3.3. Flexible and Stretchable TENG Based on In Situ Sputtered Ag

**Figure 4:**
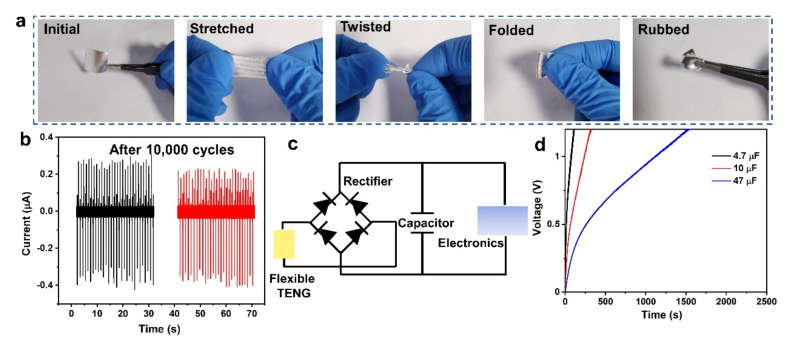
Flexible TENG and its applications. (**a**) The optical images of deformed TENG based on sputtered Ag, including stretched, folded, twisted, and rubbed states; (**b**) The performance of TENG after 10,000 cycles; (**c**) The equivalent circuit of a self-charging system that uses the energy harvested from the TENG; (**d**) Voltage curves of charging capacitors of 4.7 μF, 10 μF, and 47 μF.

With the development of wearable electronics, it is necessary to match it with a flexible, stretchable, and bendable power supplier. To examine the stability and reliability of the heterogeneous interface between in situ sputtered Ag and flexible triboelectric layer, we chose the flexible PDMS for experiments. Figure 4a includes the optical images of in situ Ag sputtered on PDMS under different deformed states (stretched, folded, twisted, and rubbed). After the physical state is severely deformed, the TENG based on sputtered Ag electrodes could be restored to its original state, and the surface of electrodes has almost no creases or damage (Figure S5, Supplementary Materials). Figure 4b is the output comparison of TENG based on sputtered Ag after 10,000 cycles. Without the protection of encapsulation layer, the output has little attenuation compared to that of the initial state, proving its excellent stability and flexibility. TENG is a capacitive conduction device, thus, the electrical energy converted from mechanical energy could be used to charge the capacitors. The circuit schematic of storing electricity manipulating output power is mainly composed of a bridge rectifier, a commercial capacitor and a dual-electrode TENG (Figure 4c). As displayed in Figure 4d, showing the different capacitors could be charged. Specifically, a capacitor of 4.7 μF is charged to 1.2 V within 112 s, whereas a larger capacitor of 10 μF is charged to 1.2 V within 323 s, furthermore, a capacitor of 47 μF is charged to 1.2 V within 1535 s, respectively, which can power various small electronics. Therefore, the flexible and stable TENG based on in situ sputtered Ag shows great potential as a reliable power supplier in the electronics.

## 4. Conclusions

In summary, we propose an in situ sputtering Ag process for fabricating induction electrodes used in TENG. Specifically, the sputtered Ag electrode and fabricated TENG are optimized by controlling the sputtering power, single-cycle time, and other parameters. In addition, the stability and applicability of sputtered Ag electrodes are investigated. It shows that under severe conditions, sputtered Ag could still maintain excellent stability, and could be used for in situ sputtering electrodes on a variety of polymer substrates. Considering the need of flexible power supplier, it is integrated with PDMS, indicating that TENG based on sputtered Ag has excellent flexibility and ductility. Finally, the developed induction electrode processing technology is used in flexible TENG and shows great prospects in self-powered electronics for practical applications. The ultimate goal of TENG is better application; therefore, the large-scale, standardized and economical fabrication of induction electrodes will aid in the development and application of TENG in the future.

## Data Availability

All data needed to evaluate the conclusions in the paper are present in the paper and/or the Supplementary Materials. Additional data related to this paper may be requested from the authors.

## Supplementary Materials

The following are available online at https://www.mdpi.com/article/10.3390/mi12101267/s1, Figure S1: The thickness of polymer films (nylon 66, PI, FEP, and PDMS) used in this work, Figure S2: The optical images of the polymer films after Ag sputtering, Figure S3: The SEM images of polymer films (nylon 66, PI, FEP, and PDMS) at ×10 K magnification, Figure S4: The testing system, including a Keithley 6514 electrometer, data acquisition card, linear motor, and TENG, Figure S5: The optical images of the sputtered Ag after severe deformation.
