# Supplementary material for: In Situ Sputtering Silver Induction Electrode for Stable and Stretchable Triboelectric Nanogenerators
Source: Micromachines (Basel). 2021 Oct 18;12(10):1267. doi: 10.3390/mi12101267 (PMC8540825; doi:10.3390/mi12101267)
Supplement: Supplementary file 1 [file micromachines-12-01267-s001.zip › micromachines-1423059-supplementary.pdf]

Supplementary Note S1: The detailed procedures for TENG examination.

The detailed motion parameters controlled by a linear motor are as follows. The used positive triboelectric layer is PET, and the area of TENG is 2×2 cm^2. The contact-separation distance is 10 mm, the acceleration of the movement is 1000 mm/s^2, and the wait time of contact is 0.1s.

Supplementary Figures:

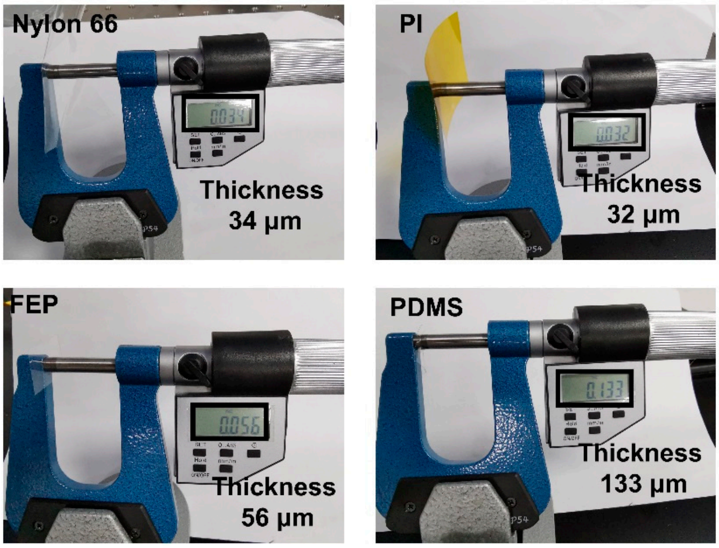

Figure S1. The thickness of the polymer films (nylon 66, PI, FEP, and PDMS) used in this work.

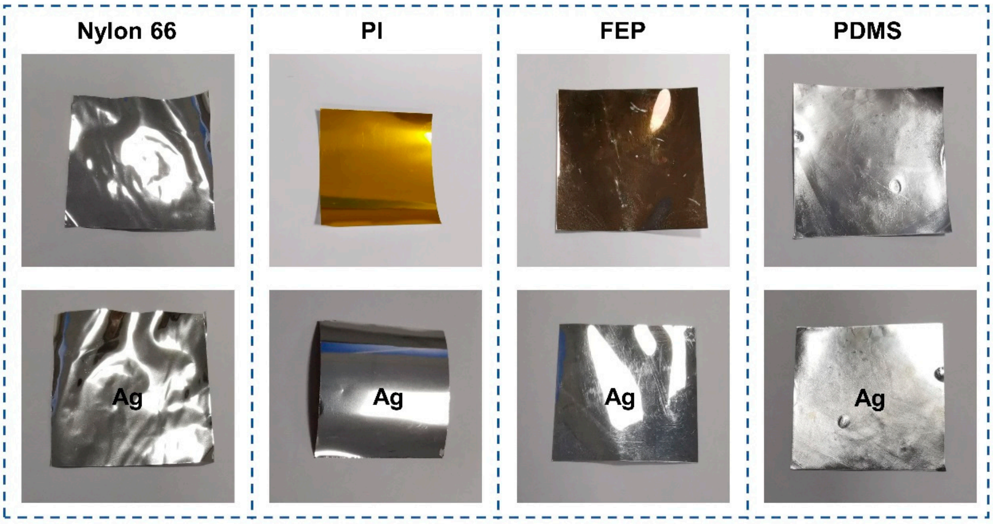

Figure S2. The optical images of the polymer films after Ag sputtering.

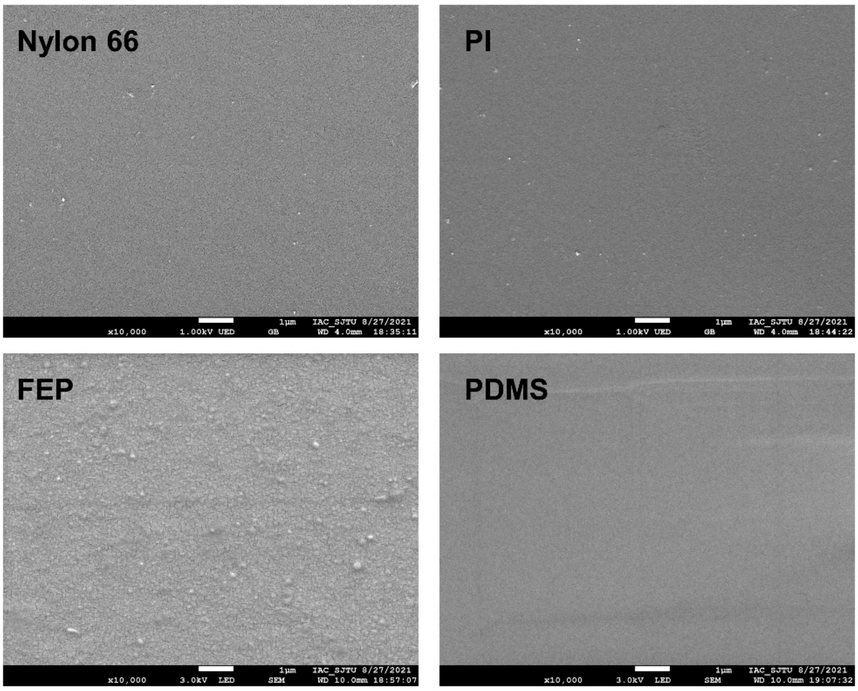

Figure S3. The SEM images of the polymer films (nylon 66, PI, FEP, and PDMS) at ×10 K magnification.

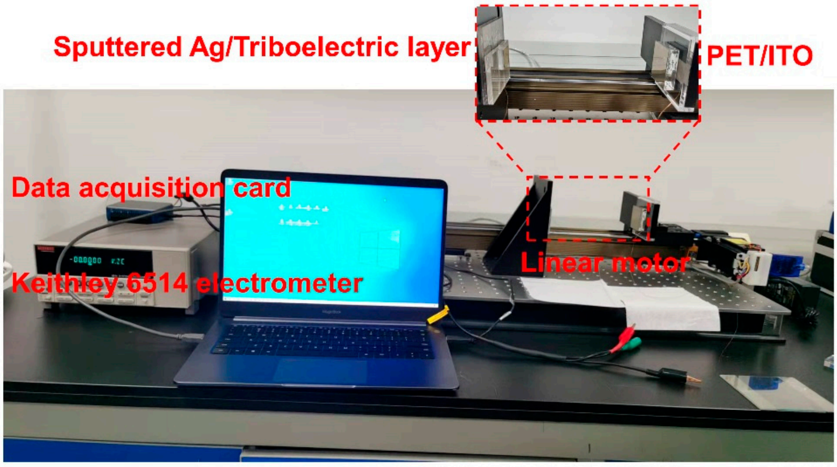

Figure S4. The testing system, including a Keithley 6514 electrometer, data acquisition card, linear motor, and TENG.

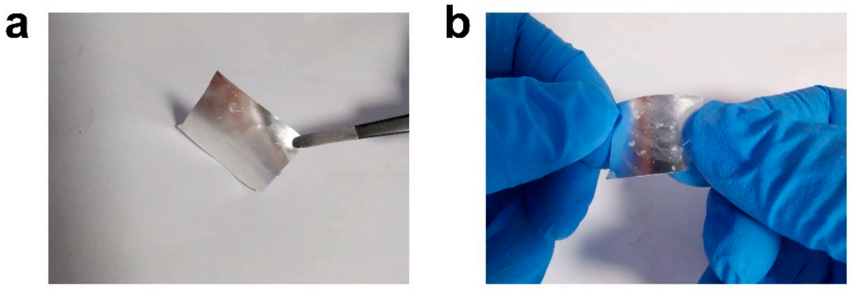

Figure S5. The optical images of the sputtered Ag after severe deformation.
